# ﻿Three new species of the primitively segmented spider genus *Songthela* (Mesothelae, Liphistiidae, Heptathelinae) from Hunan Province, China

**DOI:** 10.3897/zookeys.1154.98273

**Published:** 2023-03-17

**Authors:** Yan Zhang, Zhaoyang Chen, Daiqin Li, Xin Xu

**Affiliations:** 1 College of Life Sciences, Hunan Normal University, Changsha 410081, Hunan Province, China Hunan Normal University Changsha China; 2 Department of Biological Sciences, National University of Singapore, 14 Science Drive 4, 117543, Singapore National University of Singapore Singapore Singapore

**Keywords:** Araneae, morphology, taxonomy, trapdoor spiders

## Abstract

Three new species of the primitively segmented spider genus *Songthela* Ono, 2000 are identified and described from Hunan Province, China, based on morphological characters of males and females: *S.anhua* Zhang & Xu, **sp. nov.** (♂♀), *S.longhui* Zhang & Xu, **sp. nov.** (♂♀), and *S.zhongpo* Zhang & Xu, **sp. nov.** (♂♀). All the new *Songthela* species belong to the *multidentata*-group according to male palp and female genital morphology.

## ﻿Introduction

**Figure 1.**
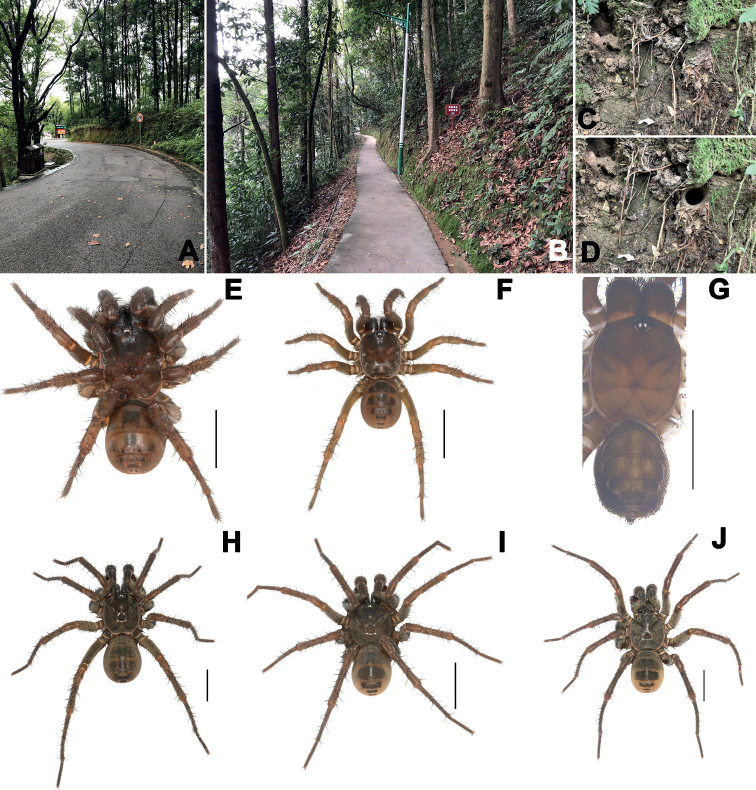
Microhabitat (**A–D**) and general somatic morphology (**E–J**) of three new *Songthela* species **E–G** female **H–J** male **E, H***Songthelazhongpo* Zhang & Xu, sp. nov. **F, I***Songthelalonghui* Zhang & Xu, sp. nov. **G, J***Songthelaanhua* Zhang & Xu, sp. nov. Scale bar: 5 mm.

The primitively segmented spider family Liphistiidae Thorell, 1869 is the basal lineage among spiders, which contains species with a limited dispersal ability and high endemicity (Bristowe 1976; Haupt 2003; Xu et al. 2015a, b). Members of liphistiids retain some plesiomorphic arachnid traits, such as abdominal tergites (Fig. 1) and spinnerets situated in the middle of abdominal venter (Bristowe 1975; Haupt 2003; Schwendinger and Ono 2011; Xu et al. 2015a). Although a few alternative taxonomic classifications for this group did exist (Kishida 1923; Haupt 1983, 2003; Ono 2000; Schwendinger and Ono 2011), the classification system of a single family Liphistiidae consisting of eight genera in two subfamilies, Liphistiinae Thorell, 1869 and Heptathelinae Kishida, 1923, was established based on the evidence from morphology, monophyly, phylogeny, and fossils (Xu et al. 2015a, b), and has been widely accepted by most arachnologists since then (Schwendinger et al. 2019; Ono and Aung 2020; Yu et al. 2021). Recently, Li (2022) elevated the two subfamilies, Liphistiinae and Heptathelinae, to family ranks, Liphistiidae and Heptathelidae, but provided no additional justification for the taxonomic classification change. Breitling (2022) argued against Li’s two-family classification system based on nomenclature, morphology, molecular data and fossils, and recommended to maintain a single family Liphistiidae. We agree with Breitling’s argumentation, and follow the classification that we have previously proposed (Xu et al. 2015a, b, 2021), which supports two subfamilies in the family Liphistiidae because all members of Liphistiidae share the same plesiomorphic traits mentioned above, although the members of Liphistiinae have signal lines around their burrow entrances, which are often used as one of the diagnostic characters to separate Liphistiinae from Heptathelinae.

Currently, the genus *Songthela* Ono, 2000, belonging to the subfamily Heptathelinae, contains 35 described species, of which, 34 are distributed in southern China (Chongqing, Guizhou, Hubei, Hunan, Sichuan, Yunnan, Zhejiang); one species, *S.sapana* (Ono, 2010) is found in northern Vietnam (World Spider Catalog 2023). Until now, 20 *Songthela* species are known from Hunan Province, and they are divided into three species groups based on morphology and molecular data: *bispina*-group, *multidentata*-group, and *unispina*-group (details see Li et al. 2022).

In this study, we diagnose and describe three new *Songthela* species collected from Hunan Province, China based on male palp and female genital morphology.

## ﻿Materials and methods

**Figure 2.**
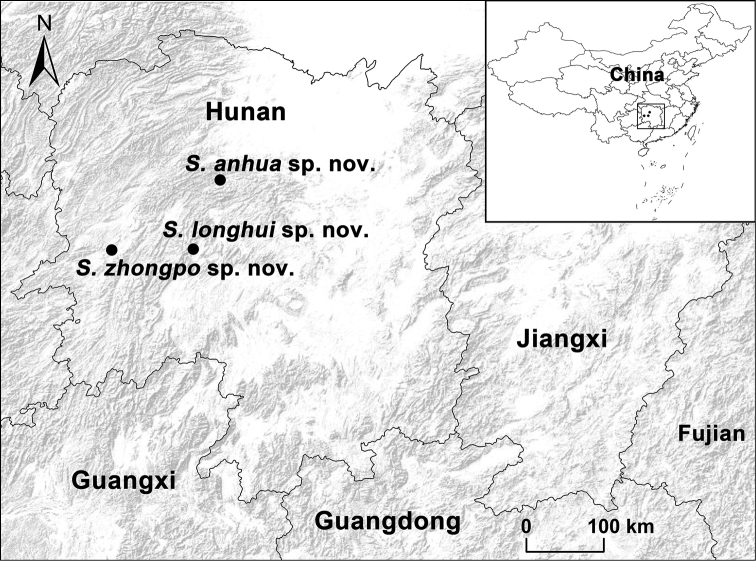
Map showing the type localities of the three new *Songthela* species in Hunan Province, China.

We collected the specimens alive from Hunan Province, China (Fig. 2), brought subadults back to the laboratory, and reared them until they reached maturation. We removed the right four legs of adults, preserved them in 100% ethanol, and stored them at –80 °C for molecular work. We preserved the remaining body of each specimen in 80% ethanol as vouchers for morphological examination. All type and voucher specimens are deposited at the College of Life Sciences, Hunan Normal University, Changsha, Hunan Province, China.

We examined and dissected the specimens using an Olympus SZ61 stereomicroscope. The soft tissues of female genitalia were removed and degraded using 10 mg/ml pancreatin (Bomei Biotech Company, Hefei, Anhui, China) for at least 3 h at the room temperature. Male palp and female genitalia were observed and photographed using the digital camera CCD mounted on an Olympus BX53 compound microscope, and then generated compound focused images using Helicon Focus v.6.7.1. All measurements are given in millimeters. Leg and palp measurements are given in the following order: leg total length (femur + patella + tibia + metatarsus + tarsus), palp total length (femur + patella + tibia + tarsus).

Abbreviations used are: **ALE** = anterior lateral eyes; **AME** = anterior median eyes; **BC** = bursa copulatrix; **BL** = body length; **CL** = carapace length; **Co** = conductor; **CT** = contrategulum; **CW** = carapace width; **DT** = dorsal extension of terminal apophysis of tegulum; **E** = embolus; **GA** = genital area; **GS** = genital stalk; **MA** = marginal apophysis of tegulum; **OL** = opisthosoma length; **OW** = opisthosoma width; **PC** = paracymbium; **PLE** = posterior lateral eyes; **PME** = posterior median eyes; **RC** = receptacular cluster; **T** = tegulum; **TA** = terminal apophysis of tegulum.

## ﻿Taxonomy

### ﻿Family Liphistiidae Thorell, 1869

**Subfamily Heptathelinae Kishida, 1923**

#### ﻿Genus Songthela

Taxon classificationAnimalia

Ono, 2000

58B8D067-03C3-5A0F-B643-770983756EFE

##### Type species.

*Heptathelahangzhouensis* Chen, Zhang & Zhu, 1981.

##### Diagnosis.

**Figure 3.**
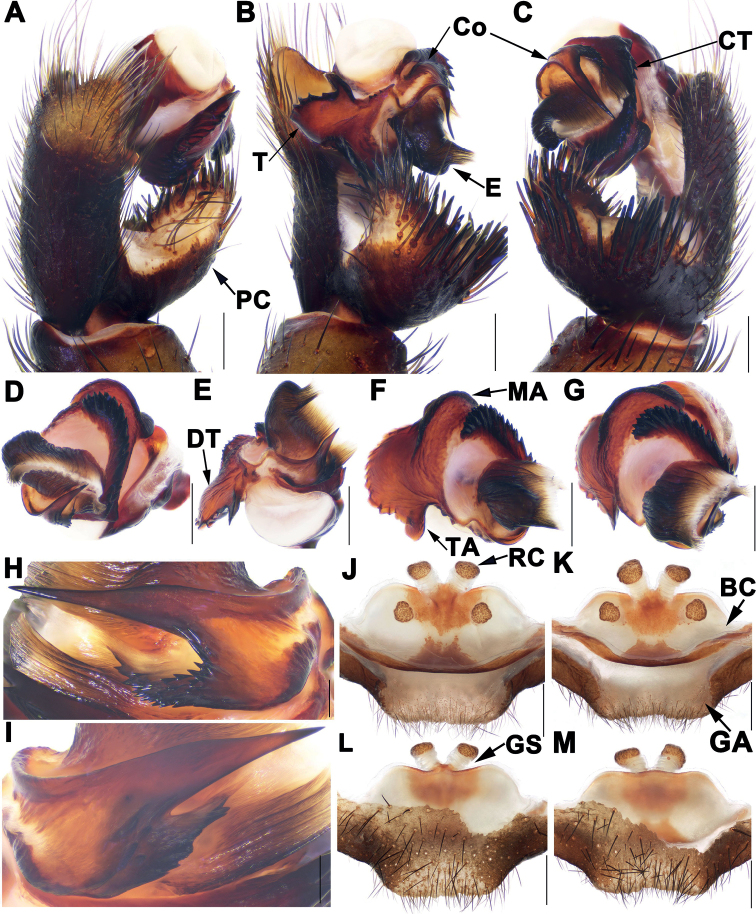
Male and female genital anatomy of *Songthelaanhua* Zhang & Xu, sp. nov. **A–C** left palp **A** prolateral view **B** ventral view **C** retrolateral view **D–G** right palp **D** prolateral view **E** ventral view **F** retrolateral view **G** distal view **H** conductor of left palp **I** conductor of right palp **J, K** vulva dorsal view **L, M** vulva ventral view **A–C, H** XUX-2021-007 (holotype) **D–G, I** XUX-2021-018 **J, L** XUX-2021-008 **K, M** XUX-2021-012. Scale bars: 0.5 mm (**A–G, J–M**); 0.1 mm (**H, I**).

**Figure 4.**
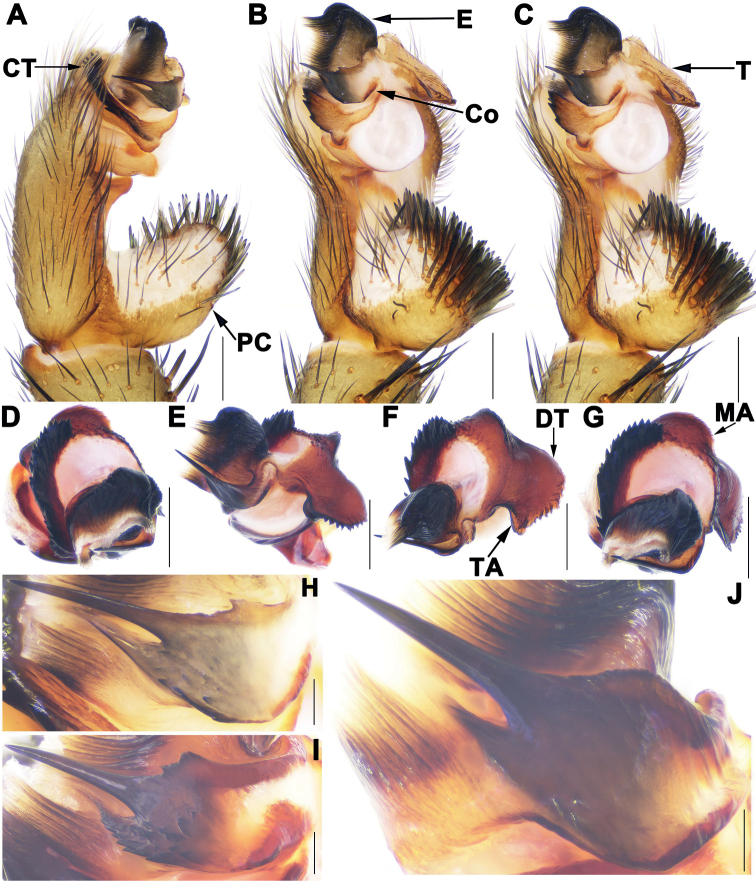
Male genital anatomy of *Songthelalonghui* Zhang & Xu, sp. nov. **A, D** palp prolateral view **B, E** palp ventral view **C, F** palp retrolateral view **G** palp distal view **H–J** conductor ventral view **A–C, H** XUX-2021-275 (holotype) **D–G, J** XUX-2021-282A **I** XUX-2021-287. Scale bars: 0.5 mm (**A–G**); 0.1 mm (**H–J**).

**Figure 5.**
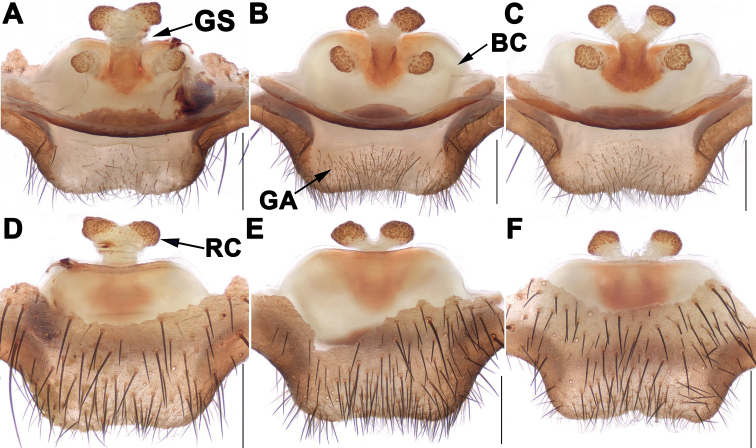
Female genital anatomy of *Songthelalonghui* Zhang & Xu, sp. nov. **A–C** vulva dorsal view **D–F** vulva ventral view **A, D** XUX-2021-281 **B, E** XUX-2021-282 **C, F** XUX-2021-278. Scale bars: 0.5 mm.

**Figure 6.**
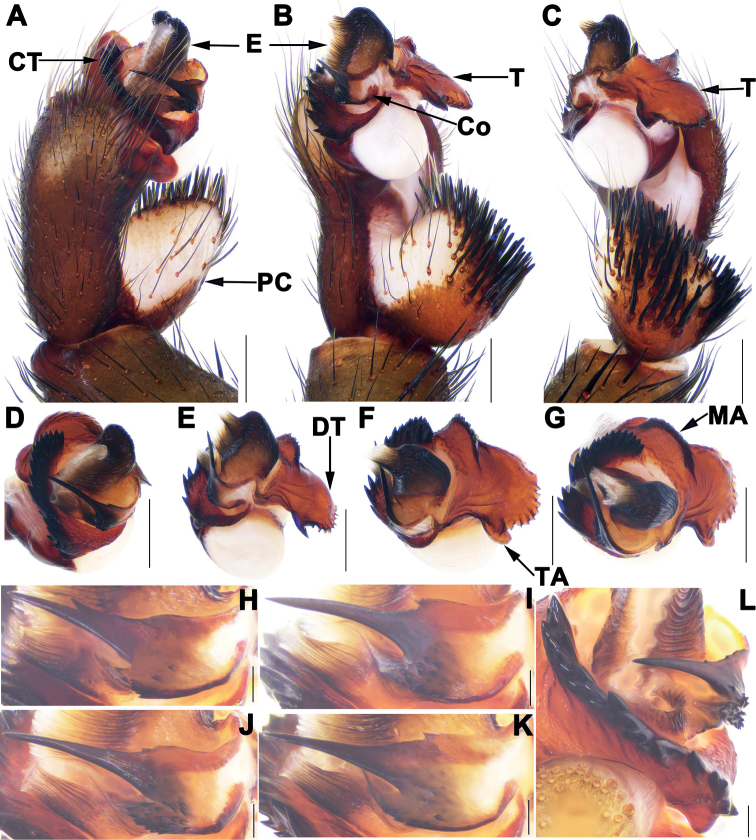
Male genital anatomy of *Songthelazhongpo* Zhang & Xu, sp. nov. **A, D** palp prolateral view **B, E** palp ventral view **C, F** palp retrolateral view **G** palp distal view **H–K** conductor ventral view **L** contrategulum prolateral view **A–C, J, L** XUX-2021-264 (holotype) **D–G, I** XUX-2021-263 **H** XUX-2021-262 **K** XUX-2021-267. Scale bars: 0.5 mm (**A–G**); 0.1 mm (**H–L**).

**Figure 7.**
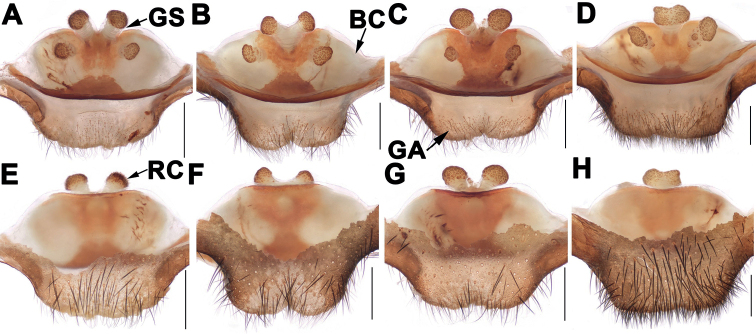
Female genital anatomy of *Songthelazhongpo* Zhang & Xu, sp. nov. **A–D** vulva dorsal view **E–H** vulva ventral view **A, E** XUX-2021-258 **B, F** XUX-2021-259 **C, G** XUX-2021-261 **D, H** XUX-2021-265. Scale bars: 0.5 mm.

Males of *Songthela* differ from those of all other Heptathelinae genera by smooth conductor with one or two apical spines and by conductor middle portion having several teeth (Figs 3B, E, 4A–C, 6A, B); by embolus having a wide and flat opening (Figs 3D, 4D, 6D); and by contrategulum having densely serrated margin (Figs 3D, 4D, 6D); females of *Songthela* can be distinguished from those of all other liphistiid genera by two pairs of receptacular clusters separated from each other, median ones with obviously tubular genital stalks, four receptacular clusters situated at the anterior margin of the bursa copulatrix, or middle ones situated at the anterior margin of the bursa copulatrix and lateral ones located relatively dorsolaterally, or all four located dorsally (Figs 3J–M, 5A–F, 7A–H).

##### Distribution.

China (Chongqing, Guizhou, Hubei, Hunan, Sichuan, Yunnan, Zhejiang) and Vietnam (Lao Cai).

#### ﻿ Songthela anhua

Taxon classificationAnimalia

Zhang & Xu sp. nov.

0190F055-35D7-51A7-ACAA-B0178136A982

https://zoobank.org/508E6FAF-B31C-4680-AFEA-7935331007AC

Fig. 3

##### Type material.

***Holotype***: China · 1 ♂; Hunan Province, Yiyang City, Anhua County, Moon Hill Park; 28.39°N; 111.22°E; alt. 125 m; 7 September 2021; Z.Y. Chen, X. Xu, Y. Zhan, Y. Zhang leg.; XUX-2021-007 (matured on 25 August 2022). ***Paratypes***: China · 1 ♂ 3 ♀; same data as for the holotype, alt. 127–144 m; XUX-2021-008, 012, 018 (matured on 25 August 2022), 019.

##### Diagnosis.

Male of *S.anhua* sp. nov. resembles those of *S.tianzhu* Chen, Li, Li & Xu, 2021, *S.yuping* Chen, Li, Li & Xu, 2021, and *S.xiangnan* Li, Liu, Li & Xu, 2020 by conductor with blade-shaped apical spine (Fig. 3C–E, H, I), but can be distinguished from *S.tianzhu* by tegulum with smaller terminal apophysis and distinctly helicoid marginal apophysis (Fig. 3F, G), by conductor with wider apical spine and several short teeth in prolateral view (Fig. 3B, C, E, H, I); from *S.xiangnan* by wider apical spine of conductor lacking of bifid apex distally (Fig. 3C, E, H, I), and by contrategulum with irregular dense dentate margin (Fig. 3C, D); from *S.yuping* by tegulum with slightly narrower dorsal extension of terminal apophysis (Fig. 3F, G); from those of *S.longhui* sp. nov. and *S.zhongpo* sp. nov. by conductor with slightly shorter and blade-shaped apical spine (Fig. 3B–E, H, I), by tegulum with slightly narrower dorsal extension of terminal apophysis (Fig. 3F, G); from those of other species of *multidentata*-group by conductor with blade-shaped apical spine (Fig. 3C–E, H, I); from those of other *Songthela* species by middle part of conductor with several short teeth (Fig. 3B–E, H, I).

Females of *S.anhua* sp. nov. can be distinguished from those of *S.longhui* sp. nov. and *S.pluma* Yu, Li & Zhang, 2018 by median receptacular clusters slightly larger than lateral ones, and middle genital stalks separated from each other basally (Fig. 3J, K); from *S.zhongpo* sp. nov. by trapeziform anterior margin of bursa copulatrix, median receptacular clusters with slightly longer genital stalks (Fig. 3J–K); from those of other species of *multidentata*-group by median receptacular clusters separated from each other basally and posterior margin of genital area wider and flat (Fig. 3J–M); from those of other *Songthela* species by four receptacular clusters situated at the dorsal side of bursa copulatrix (Fig. 3J–M).

##### Description.

**Male** (holotype; Fig. 1J). Carapace black brown; opisthosoma yellow brown, with 12 black brown tergites attached a pair of thick bristles, the second to fifth larger than others and the fourth largest; sternum narrow, much longer than wide; a few pointed hairs running over ocular area; chelicerae robust with promargin of cheliceral groove with 14 denticles of variable size; legs with sturdy hairs and spines; 8 spinnerets. Measurements: BL 13.28, CL 6.42, CW 5.70, OL 6.19, OW 4.85; ALE > PLE > PME > AME; leg I 20.58 (5.80 + 2.53 + 4.25 + 5.27 + 2.73), leg II 19.69 (5.54 + 2.52 + 4.18 + 4.88 + 2.57), leg III 22.95 (5.56 + 2.69 + 4.28 + 6.95 + 3.47), leg IV 28.29 (7.32 + 3.03 + 5.69 + 7.97 + 4.28).

***Palp*.** Prolateral portion of paracymbium unpigmented and unsclerotised, with several setae and spines on the tip (Fig. 3A–C). Contrategulum with an obviously triangular apophysis proximally and two irregular dentate edges distally (Fig. 3C, D). Tegulum with a helicoid marginal apophysis, a dentate dorsal extension of the terminal apophysis, and a thumb-like terminal apophysis retrolaterally (Fig. 3F, G). Conductor lamellar, fused with embolus ventroproximally, with a blade-shaped apical spine pointed to the one-third of opening of embolus proximally, and the middle portion inserted with several teeth (Fig. 3B–E, H, I). Embolus largely sclerotized with a wide opening, several ribbed ridges, and with a twisted top in ventral view (Fig. 3B–E, G).

**Female** (XUX-2021-008; Fig. 1G). Carapace yellow brown and opisthosoma dark brown in alcohol, with 12 dark brown tergites attached a pair of thick bristles, the second to fifth larger than others and the fourth largest; sternum narrow, much longer than wide; a few pointed hairs running over ocular area; chelicerae robust with promargin of cheliceral groove with 12 denticles of variable size; legs with sturdy hairs and spines; 7 spinnerets. Measurements: BL 10.18, CL 5.16, CW 4.52, OL 4.43, OW 3.57; ALE > PLE > PME > AME; palp 9.82 (3.37 + 1.81 + 2.41 + 2.23), leg I 10.97 (3.66 + 1.83 + 2.16 + 1.96 + 1.36), leg II 10.29 (3.21 + 1.79 + 1.86 + 2.06 + 1.37), leg III 9.71 (2.91 + 1.74 + 1.22 + 2.27 + 1.57), leg IV 14.37 (4.22 + 1.60 + 2.49 + 3.72 + 2.34).

***Female genitalia*.** Two pairs of receptacular clusters situated on the dorsal side of the bursa copulatrix; the middle pair of receptacular clusters with long genital stalks and larger than the lateral ones, the middle stalks separated from each other; the posterior margin of the bursa copulatrix sclerotized; the posterior margin of the genital area wide (Fig. 3J–M).

##### Variation.

Males and females vary in body size, cheliceral teeth, and spinnerets. Range of measurements in males (*N* = 2): BL 11.94–13.28, CL 5.59–6.42, CW 5.07–5.70, OL 6.08–6.19, OW 4.80–4.85; the number of cheliceral teeth varies from 9–14 (*N* = 2); there are 7 or 8 spinnerets. Females (*N* = 3): BL 10.18–11.51, CL 5.16–5.57, CW 4.52–4.84, OL 4.43–5.66, OW 3.57–4.04; the number of cheliceral teeth varies from 12–13 (*N* = 3); there are 7 or 8 spinnerets. In addition, male palp and female genitalia also show intraspecific variations: in males, the left palp is slightly different from the right palp, e.g. the tegulum of left male palp with three teeth basally in ventral view (Fig. 3B), but they are missing in right male palp (Fig. 3E, F); the number of teeth located in middle portion of conductor also varies between left and right palp (Fig. 3C, E, H, I); in females, the relative position of the middle receptacular clusters situated on the dorsal side of the bursa copulatrix is slightly different (Fig. 3L, M).

##### Etymology.

The species epithet, a noun in apposition, refers to the type locality.

##### Distribution.

Hunan (Anhua), China

#### ﻿ Songthela longhui

Taxon classificationAnimalia

Zhang & Xu sp. nov.

4972EC6B-DFB1-568A-9607-65B6A6FA005E

https://zoobank.org/EBBC224B-1404-41B6-B08B-EC8D3A5749AB

Figs 4 , 5

##### Type material.

***Holotype***: China · 1 ♂; Hunan Province, Shaoyang City, Longhui County, Jinshiqiao Town, Huangjinjing Village; 27.58°N; 110.90°E; alt. 550 m; 18 September 2021; Z.Y. Chen, X. Xu, Y. Zhan, Y. Zhang leg.; XUX-2021-275 (matured on 25 August 2022). ***Paratypes***: China · 1 ♂, 5 ♀; same data as for the holotype, alt. 552 m; XUX-2021-278, 281, 282, 282A (matured on 25 August 2022), 283, 285A.

##### Diagnosis.

Male of *S.longhui* sp. nov. resembles those of *S.dapo* Li, Chen, Liu, Li & Xu, 2022, *S.lingshang* Li, Chen, Liu, Li & Xu, 2022, *S.multidentata* Li, Chen, Liu, Li & Xu, 2022, *S.pluma* and *S.xiujian* Li, Chen, Liu, Li & Xu, 2022 by conductor with needle-shaped apical spine (Fig. 4A, B, E, H–J), but can be distinguished from those of *S.dapo* and *S.lingshang* by tegulum with smaller dorsal extension of terminal apophysis (Fig. 4C, F), and conductor with slightly narrower base of apical spine (Fig. 4A, H–J); from *S.multidentata* by conductor with longer apical spine (Fig. 4A, B, E, H–J), and contrategulum with larger apophysis proximally (Fig. 4A, B, D); from *S.pluma* by tegulum with smaller terminal apophysis (Fig. 4F), and contrategulum with one irregular dentate margin (Fig. 4A, D); from *S.xiujian* by contrategulum with larger apophysis proximally (Fig. 4A, B, D); from *S.anhua* sp. nov. by apical spine of conductor needle-shaped (Fig. 4A, B, H–J), by tegulum with slightly smaller terminal apophysis and wider dorsal extension of terminal apophysis (Fig. 4C, F); from *S.zhongpo* sp. nov. by apical spine of conductor with slightly narrower base (Fig. 4A, H–J); from those of other species of *multidentata*-group by needle-shaped apical spine of conductor (Fig. 4A, E, H–J); from those of other *Songthela* species by middle part of the conductor with several small spines (Fig. 4A, B, E, H–J).

Females of *S.longhui* sp. nov. can be distinguished from *S.anhua* sp. nov. by Y-shaped median genital stalks, lateral receptacular clusters with distinct short genital stalks, and deeper depressions in dorsal view (Fig. 5A–C); from *S.pluma* by lateral receptacular clusters with slightly longer genital stalks, and two larger and deeper depressions in dorsal view (Fig. 5A–C); from *S.zhongpo* sp. nov. by median receptacular clusters with longer genital stalks (Fig. 5A–C); from those of other species of *multidentata*-group by median receptacular clusters with longer genital stalks, and Y-shaped median genital stalks, lateral ones with distinct genital stalks (Fig. 5A–C); from those of other *Songthela* species by four receptacular clusters located at dorsal side of bursa copulatrix and median genital stalks fused together basally (Fig. 5A–C).

##### Description.

**Male** (holotype). Carapace brown; opisthosoma yellow brown, with 12 brown tergites attached a pair of hard and thick bristles, the second to fifth larger than others and the fourth largest; sternum narrow, much longer than wide; a few pointed hairs running over ocular area; chelicerae robust with promargin of cheliceral groove with 11 denticles of variable size; legs with sturdy hairs and spines; 6 spinnerets. Measurements: BL 11.94, CL 5.36, CW 4.63, OL 5.91, OW 4.72; ALE > PLE > PME > AME; leg I 16.52 (4.72 + 2.10 + 3.32 + 4.05 + 2.33), leg II 16.34 (4.37+ 2.09 + 3.13 + 4.23 + 2.52), leg III 19.10 (5.17 + 2.07 + 3.37 + 5.47 + 3.02), leg IV 23.98 (5.80 + 2.44 + 4.65 + 7.31 + 3.78).

***Palp*.** Paracymbium unpigmented and unsclerotised prolaterally, numerous setae and spines on the tip (Fig. 4A–C). Contrategulum with an arched apophysis proximally and irregular dentate edge (Fig. 4A, B, D). Tegulum with a semi-circular marginal apophysis and dentate dorsal extension of the terminal apophysis, and with a small terminal apophysis retrolaterally (Fig. 4C, F, G). Conductor having a long apical spine pointed to the one-third of opening of embolus proximally, the middle part covered with several small teeth, and the smooth base fused with embolus (Fig. 4A, B, E, H–J). Embolus largely sclerotized, with a wide and flat opening, several longitudinal ribs in middle and distal portion (Fig. 4A, B, D, E, G).

**Female** (XUX-2021-281; Fig. 1F). Carapace dark reddish brown and opisthosoma light brown, with 12 dark brown tergites attached a pair of thick bristles, the second to fifth larger than others and the fourth largest; sternum narrow, much longer than wide; a few pointed hairs running over ocular area; chelicerae robust with promargin of cheliceral groove with 12 denticles of variable size; legs with sturdy hairs and spines; 7 spinnerets. Measurements: BL 11.52, CL 5.20, CW 4.33, OL 5.73, OW 4.51; ALE > PLE > PME > AME; palp 9.41 (3.36 + 1.56 + 1.90 + 2.59), leg I 10.69 (3.41 + 1.72 + 2.12 + 2.03 + 1.41), leg II 10.56 (3.29 + 1.76 + 1.82 + 2.14 + 1.55), leg III 11.23 (3.22 + 1.80 + 1.97 + 2.58 + 1.66), leg IV 15.96 (4.56 + 2.07 + 2.82 + 4.22 + 2.29).

***Female genitalia*.** Two pairs of receptacular clusters with distinctly genital stalks, situated on the dorsal wall of the bursa copulatrix; the median ones similar to or slightly larger than the lateral ones, the Y-shaped middle genital stalks; the posterior margin of the bursa copulatrix sclerotized, the posterior margin of the genital area wide, two deeper depressions in dorsal view (Fig. 5A–F).

##### Variation.

Males and females vary in body size, cheliceral teeth and spinnerets. Range of measurements in males (*N* = 2): BL 10.98–11.94, CL 4.95–5.36, CW 4.52–4.63, OL 5.60–5.91, OW 4.22–4.72. There are 6 or 7 spinnerets (*N* = 2). Females (*N* = 5): BL 5.56–11.86, CL 4.27–5.48, CW 3.61–4.58, OL 4.29–5.79, OW 3.27–4.60. The number of cheliceral teeth varies from 12 to 13 (*N* = 5). In addition, male palp and female genitalia also show intraspecific variations: in males, the middle part of conductor with more teeth (Fig. 4H, I) or less teeth (Fig. 4J); tegulum with a relatively larger terminal apophysis (Fig. 4C) or slightly smaller (Fig. 4E). In females, the middle Y-shaped genital stalk fused totally with only two receptacular clusters separated from each other (Fig. 5A, D) or fused basally and separated in the middle (Fig. 5B, C, E, F).

##### Etymology.

The species epithet, a noun in apposition, refers to the type locality.

##### Distribution.

Hunan (Longhui), China

#### ﻿ Songthela zhongpo

Taxon classificationAnimalia

Zhang & Xu sp. nov.

804B48B9-C09D-5502-9232-FF9780BA1674

https://zoobank.org/A5692990-DD85-4BDA-BE44-953DED723609

Figs 6 , 7

##### Type material.

***Holotype***: China · 1 ♂; Hunan Province, Huaihua City, Hecheng District, Zhongpo Forest Park; 27.57°N; 110.96°E; alt. 330 m; 17 September 2021; Z.Y. Chen, X. Xu, Y. Zhan, Y. Zhang leg.; XUX-2021-264 (matured on 3 August 2022). ***Paratypes***: China · 3 ♂ 9 ♀; same data as for the holotype, alt. 300–345 m; XUX-2021-258, 259, 260, 261, 262 (matured on 18 September 2022), 263 (matured on 20 July 2022), 265, 266, 267 (matured on 26 July 2022), 267A, 267B, 267C.

##### Diagnosis.

Male of *S.zhongpo* sp. nov. resembles those of *S.dapo*, *S.lingshang*, and *S.xiujian*, by apical spine of conductor with slightly wider base (Fig. 6A, D, E, H–K), but can be distinguished from *S.dapo* by tegulum with smaller terminal apophysis (Fig. 6C, F, G); from *S.lingshang* by contrategulum with slightly larger teeth (Fig. 6D, F, G, L), conductor with fewer teeth in middle part (Fig. 6A, D, E, H–K), and tegulum with a small terminal apophysis (Fig. 6C, F, G); from *S.xiujian* by tegulum with arched marginal apophysis (Fig. 6G), and contrategulum with slightly smaller apophysis proximally (Fig. 6A, L); from *S.anhua* sp. nov. by apical spine of conductor narrower and longer (Fig. 6A, B, D, H–K); from *S.longhui* sp. nov. by apical spine of conductor with slightly narrower base (Fig. 6A, H–K), and surface of dorsal extension of the tegular terminal apophysis with several ridges (Fig. 6G); from those of other species of *multidentata*-group by contrategulum with two dentate margins in middle part (Fig. 6D, L), by apical spine of conductor with slightly wider base and gradually becomes elongated distally (Fig. 6A, D, E, H–K); from those of other *Songthela* species by middle part of the conductor with several teeth (Fig. 6A, B, D, E, H–K).

Female of *S.zhongpo* sp. nov. can be distinguished from *S.anhua* sp. nov. by the lateral receptacular clusters with slightly longer genital stalks, and arched anterior margin of bursa copulatrix (Fig. 7A–D); from *S.longhui* sp. nov. by median receptacular clusters with shorter genital stalks (Fig. 7A–D); from *S.multidentata* by median receptacular clusters with thicker genital stalks (Fig. 7A–D), from *S.tianzhu* by lateral genital stalks slightly longer (Fig. 7A, D); from those of other species of *multidentata*-group by median receptacular clusters with slightly thicker genital stalks, and lateral ones with distinct genital stalks (Fig. 7A–H); from those of other *Songthela* species by two pairs of receptacular clusters situated on dorsal wall of bursa copulatrix (Fig. 7A–H).

##### Description.

**Male** (holotype). Carapace black brown; opisthosoma dark brown, with 12 brown tergites attached a pair of thick bristles, the second to fifth larger than others and the fourth largest; sternum narrow, much longer than wide; a few pointed hairs running over ocular area; chelicerae robust with promargin of cheliceral groove with 11 denticles of variable size; legs with sturdy hairs and spines; 7 spinnerets. Measurements: BL 13.53, CL 5.96, CW 5.44, OL 7.19, OW 5.94; ALE > PLE > PME > AME; leg I 18.97 (5.30 + 2.29 + 3.95 + 4.93 + 2.50), leg II 19.13 (4.99 + 2.33 + 3.93 + 5.24 + 2.64), leg III 21.49 (5.25 + 2.46 + 3.91 + 6.52 + 3.35), leg IV 27.19 (6.62 + 2.84 + 5.17 + 8.59 + 3.97).

***Palp*.** Paracymbium unpigmented and unsclerotised prolaterally, with several setae and spines on the tip (Fig. 6A–C). Contrategulum with a triangular apophysis proximally and with a dentate edge in distal and proximal portions, while with two dentate margins in the middle part (Fig. 6A, D, F, G, H–K). Tegulum with an arched helicoid marginal apophysis, a helicoid dorsal extension of the terminal apophysis, and a thumb-shaped terminal apophysis retrolaterally (Fig. 6C, E–G). Conductor fused with embolus basally and having several teeth in the middle part, the long apical spine with a spinule basally and pointed to the one-fourth of opening of embolus proximally (Fig. 6A, B, D–K). Embolus largely sclerotized, with a wide and flat opening, numerous longitudinal ribs in middle and distal portion (Fig. 6B, D–F).

**Female** (XUX-2021-258). Carapace reddish brown and opisthosoma light brown, with 12 dark brown tergites attached a pair of thick bristles, the second to fifth larger than remaining ones and the fourth largest; sternum narrow, much longer than wide; a few pointed hairs running over ocular area; chelicerae robust with promargin of cheliceral groove with 11 denticles of variable size; legs with sturdy hairs and spines; 7 spinnerets. Measurements: BL 9.32, CL 4.62, CW 3.95, OL 3.93, OW 3.31; ALE > PLE > PME > AME; palp 8.57 (3.05 + 1.56 + 1.84 + 2.12), leg I 9.17 (2.57 + 1.79 + 1.82 + 1.76 + 1.23), leg II 8.57 (1.96 + 1.65 + 1.77+ 1.84 + 1.35), leg III 9.72 (2.62 + 1.58 + 1.74 + 2.27 + 1.51), leg IV 14.28 (4.07 + 1.90 + 2.67 + 3.61 + 2.03).

***Female genitalia*.** Four receptacular clusters situated on the dorsal side of the bursa copulatrix; the middle ones with thick genital stalks close to each other, fused together basally and separated from each other distally (Fig. 7A–C), or the middle genital stalks fused as one (Fig. 7D); the lateral receptacular clusters similar to or smaller than the middle ones; the posterior margin of the bursa copulatrix sclerotized, the posterior margin of the genital area wide and straight (Fig. 7A, D), or incurved in the middle (Fig. 7B, C).

##### Variation.

Males and females vary in body size, cheliceral teeth and spinnerets. Range of measurements in males (*N* = 4): BL 12.54–13.80, CL 4.96–6.09, CW 5.29–5.89, OL 6.02–7.19, OW 4.23–5.94. The number of cheliceral teeth varies from 11 to 13. There are 6 or 8 spinnerets (*N* = 4). Females (*N* = 9): BL 9.32–13.77, CL 4.62–6.40, CW 3.95–5.49, OL 3.93–6.32, OW 3.31–5.14. The number of cheliceral teeth varies from 10 to 12. There are 7 or 8 spinnerets (*N* = 9). In addition, male palp and female genitalia also show intraspecific variations: in males, the apical spine of conductor with a spinule in the middle part (Fig. 6H) or basally (Fig. 6A, D, I–K); the middle of conductor with more teeth (Fig. 6I, L) or relatively few teeth (Fig. 6H, K). In females, the middle pair of receptacular clusters similar (Fig. 7B) to or slightly larger than the lateral ones (Fig. 7A, C), the middle genital stalks fused together basally and separated from each other distally (Fig. 7A, C), or the middle genital stalks Y-shaped (Fig. 7B), or fused together totally (Fig. 7D).

##### Etymology.

The species epithet, a noun in apposition, refers to the type locality.

##### Distribution.

Hunan (Huaihua), China

##### Remarks.

The three new species from Hunan Province, China can be assigned into the *multidentata*-group based on the following characters of both male palp and female genital morphology: 1) conductor of male palp with one apical spine and the middle part covered several teeth; 2) female genitalia with two pairs of receptacular clusters situated on the dorsal side of the bursa copulatrix; and 3) the posterior margin of the bursa copulatrix of female genitalia pigmented and sclerotised.

## Supplementary Material

XML Treatment for Songthela

XML Treatment for Songthela anhua

XML Treatment for Songthela longhui

XML Treatment for Songthela zhongpo
